# Fixed-Bed Adsorption of Lead from Aqueous Solution Using Chitosan-Coated Bentonite

**DOI:** 10.3390/ijerph19052597

**Published:** 2022-02-23

**Authors:** Cybelle Morales Futalan, Meng-Wei Wan

**Affiliations:** 1 Department of Community and Environmental Resource Planning, University of the Philippines, Los Banos 4031, Philippines; cmfutalan@up.edu.ph; 2 Department of Environmental Engineering and Science, Chia Nan University of Pharmacy and Science, Tainan 71710, Taiwan

**Keywords:** adsorption, bentonite, breakthrough curve, Clark model, chitosan, fixed-bed, lead, Thomas model

## Abstract

In this study, fixed-bed adsorption of Pb(II) from an aqueous solution using chitosan-coated bentonite (CCB) was investigated. Characterization of CCB was performed using Fourier transform infrared spectroscopy (FT-IR) and scanning electron microscopy (SEM). The effects of varying bed height (1.3 to 4.3 cm), flow rate (0.20 to 0.60 mL/min), and initial concentration (500 to 1500 mg/L) on the length of mass transfer zone (*Z_m_*) and adsorption capacity at breakthrough (*q_b_*) and exhaustion (*q_e_*) were examined. Low flow rate and high bed height were determined to cause a longer time to reach breakthrough and exhaustion. Meanwhile, the fixed-bed system was observed to quickly attain breakthrough and exhaustion under high initial concentrations. Kinetic column models such as the Thomas, Yoon–Nelson, and Clark models were used to predict the breakthrough curves. High *R*^2^ values (0.9758 ≤ *R*^2^ ≤ 0.8087) were attained for the Thomas model, which indicates that there is good agreement between experimental data and linear plots generated by the Thomas model. Moreover, the Thomas model is best in describing the breakthrough curves of Pb(II) removal under a fixed-bed system.

## 1. Introduction

Heavy metal contamination in water bodies has always been a critical environmental concern worldwide. Lead, Pb(II), is known to be one of the most toxic heavy metals that is nonbiodegradable and could bioaccumulate in the environment [1,2]. It is found to be the third most frequently occurring heavy metal pollutant that is present in groundwater and surface waters [3]. Pb(II) in waste streams are mainly generated by several anthropogenic activities including mining, ammunition and explosives manufacturing, the ceramic industry, e-wastes, paint manufacturing, and battery production [4,5]. Globally, about 783,000 MT of Pb(II) is released into the environment on an annual basis [6]. The discharge of untreated wastewater containing Pb(II) is detrimental to both the environment and human health. Diseases such as anemia, damage to the kidney and nervous system, mental retardation, cardiovascular disease, cognitive impairment, and infertility in men have been associated with long-term exposure to Pb(II) [7]. High accumulation of Pb(II) in plants has negative effects such as stunted plant growth, decreased CO_2_ fixation capacity, and lower production of leaves and biomass [8,9]. Hence, the maximum contaminant level of Pb(II) in drinking water has been fixed at 0.015 mg/L by the United States Environmental Protection Agency [10]. Based on the Philippine National Standard of Drinking Water, the permissible limit for Pb(II) is set at 0.01 mg/L [11].

Adsorption is one of the oldest wastewater treatment technologies that is commonly utilized due to its simple operation, capability for adsorbent regeneration, cost-effectiveness, and high efficiency [12]. In addition, it also has the ability to process a high volume of wastewater minus the production of noxious sludge [13]. Low-cost and environmentally friendly adsorbents such as chitosan [14,15], clay materials [16,17,18], rice husk ash [19], spent coffee ground [20], crab shells [21], and tea leaves [22] have recently gained attention as alternatives to commercial adsorbents.

Chitosan is a linear, polycationic biopolymer that is formed via deacetylation of chitin under alkaline conditions. The chitosan structure is composed of *N*-acetyl-d-glucosamine units connected by β(1→4) glycosidic bonds [23]. Chitin is derived from shrimp shells produced by seafood processing plants as waste products [24]. The wide application of chitosan in wastewater treatment, medicine, and different industries can be attributed to its attractive properties such as hydrophilicity, capacity for chelation of heavy metals, non-toxicity, and biodegradability [25]. Chitosan has the ability to form chelated complexes, hydrogen bonds, and electrostatic bonds with water contaminants due to the numerous functional groups, such as amino and hydroxyl groups, found on its macromolecular linear backbone [26,27]. However, pure chitosan has low surface area, is mechanically and thermally unstable, easily dissolves in slightly acidic media, and is brittle in nature [28,29]. Modification of chitosan, whether chemical or physical in nature, has been performed to overcome these disadvantages. Bentonite is a non-metallic clay mineral with a unit cell structure of 2:1 that is composed of two silica tetrahedral sheets and one alumina octahedral sheet located between the tetrahedral sheets [30]. Bentonite is widely utilized in wastewater treatment facilities due to its environmentally friendly nature, mechanical stability, availability, and low cost [31,32]. It also has a high removal capacity for organic and inorganic pollutants, which can be attributed to its excellent cation exchange capacity and high surface area [31,33].

Hybrid materials such as polymer–clay composites have shown to be a promising alternative adsorbent material in wastewater treatment. Polymer–clay composites have displayed enhanced properties such as improved surface area, thermal and chemical stability, heat resistance, and mechanical strength [34]. Literature review shows numerous studies on polymer–clay composites and their environmental applications. Rusmin et al. [10] developed the magnetic chitosan–palygorskite nanocomposite with an adsorption capacity of 58.5 mg/g in removing lead from aqueous solution. Porous magnetic bentonite–chitosan beads were determined to have a maximum adsorption capacity of 57.1 mg/g in removing radioactive cesium from synthetic solution [35]. Chitosan–montmorillonite nanocomposite was developed to remove Congo Red dye in water [36]. Chitosan-coated bentonite (CCB) was used in the removal of dibenzothiophene sulfone, In(III), Cu(II), Ni(II), Pb(II), As(V), ammonium, and chemical oxygen demand [37,38,39,40,41,42,43,44,45,46]. However, most of the studies performed on chitosan–clay composites in the removal of heavy metals and organic contaminants were conducted under batch conditions. In real-life applications, batch systems would require a large amount of adsorbent to process a high volume of wastewater under a shorter time period. Meanwhile, column or fixed-bed studies would provide data for practical applications such as the remediation of groundwater via the permeable reactive barrier (PRB) system. The results of the present work could be utilized in examining the possibility of chitosan-coated bentonite (CCB) as a reactive material in a PRB system. A typical PRB is installed by either excavating a shallow trench, which would be backfilled with reactive materials, or injected directly into the aquifer that is located at a depth greater than 50 ft. A PRB is placed perpendicular to the flow direction of the contaminated groundwater plume [47]. Previous studies have investigated the removal of chlorinated contaminants, heavy metals, organic contaminants, and radionuclides using reactive materials such as zero valent iron, zeolite, limestone, carbonized food waste, and activated carbon [47,48,49]. The permeability of PRB is maintained by mixing reactive material with sand and the contaminated plume is treated via adsorption, oxidation–reduction reaction, or precipitation as it passes through the reactive material. The synthesis of the CCB composite material implies that a lower quantity of chitosan will be used since bentonite is a geologically available material [37]. The study of Tsai et al. [40] investigated the competitive removal of Pb(II), Ni(II), and Cu(II) using CCB in a multi-metal system. So far, there are no studies on CCB as an adsorbent for Pb(II) removal under dynamic conditions in a single-metal solution.

In continuation of the studies of Futalan et al. [37] and Tsai et al. [40], the present work aims to assess the performance of CCB and its adsorptive removal of Pb(II) under fixed-bed conditions. The effects of design parameters including flow rate, initial concentration, and bed height on the shape of the breakthrough curve and breakthrough time were investigated. Kinetic models such as the Clark, Yoon–Nelson, and Thomas models were used to examine the experimental data and evaluate the fixed-bed performance.

## 2. Materials and Methods

### 2.1. Chemicals

Bentonite, Pb(NO_3_)_2_, and NaNO_3_ were procured from Riedel-de Haën (USA). Chitosan powder (75–85% deacetylation degree, low molecular weight) was obtained from Sigma-Aldrich (Selangor D.E, Petaling Jaya, Malaysia). ICP standard multi-element solution, NaOH (99% purity) pellets, and HCl (37% fuming) were acquired from Merck (Darmstadt, Germany). All chemicals used were of analytical grade.

### 2.2. Synthesis of Chitosan-Coated Bentonite

In a 1 L beaker, the dissolution of 5 g chitosan in 300 mL of 5% (*v*/*v*) HCl solution was performed under constant stirring using a magnetic plate (PC-420D CORNING, New York, NY, USA) at 300 rpm for 2 h. Subsequently, bentonite (100 g) was slowly added, and the mixture was continuously stirred for 3 h. The mixture was neutralized with 1 M NaOH in a drop-wise manner. Unreacted material was removed by washing the adsorbent beads with deionized water several times. Afterwards, the adsorbent was dried in an oven (Channel Precision Oven DV452 220 V, Yantai, China) for 24 h at 65 °C. The adsorbent was then ground and sieved using a 200 µm mesh screen. The prepared adsorbent is referred to as chitosan-coated bentonite beads (CCB) with particle size ranging from 0.50 mm to 0.21 mm.

### 2.3. Characterization of CCB

The functional groups of CCB before and after adsorption were examined using Fourier transform infrared spectroscopy (FT-IR, Jasco 410, Easton, MD, USA) using the KBr pellet technique in the range of 400 to 4000 cm^−1^ with 8 cm^−1^ resolution. Scanning electron microscopy (Hitashi S-4800 SEM, Tokyo, Japan) was utilized to determine the surface morphology of the adsorbent.

### 2.4. Fixed-Bed Experiments

Fixed-bed studies were performed using a glass column (IWAKI borosilicate glass, West Java, Indonesia) with length and inner diameter of 55 cm and 1.9 cm, respectively. At the top of the CCB bed, glass beads and sand were placed to ensure effluent flow is evenly distributed as it enters the fixed bed. To avoid washing out the CCB adsorbent, glass beads and sand were positioned at the bottom of the column. The influent was fed at the top of the column and operated in a downflow mode using a peristaltic pump (Masterflex CZ 77120-70, Selangor D.E, Petaling Jaya, Malaysia). The effects of initial concentration (500 to 1500 mg/L), flow rate (0.20 to 0.60 mL/min), and bed height (1.3 to 4.3 cm) on the shape of the breakthrough curve and breakthrough capacity were examined. Samples were collected at the bottom of the column at pre-determined time intervals. The collected effluent was filtered using a 0.45 µm Whatman filter, preserved through acidification using 1 N HNO_3_ and stored at 4 °C prior to analysis. The residual concentration of Pb(II) was measured using an inductively coupled plasma optical emission spectrometer (ICP-OES, Perkin Elmer DV 2000 series, Waltham, MA, USA).

### 2.5. Analysis of Fixed-Bed Data

The shape of the breakthrough curve indicates the performance of the fixed-bed study. The breakthrough curve is plotted as *C_t_*/*C*_0_ against time. Variables *C*_0_ and *C_t_* refer to the initial concentration and effluent concentration at any time *t*, respectively. The exhaustion time and breakthrough time were defined as the concentration of treated effluent when it reaches 90% and 10% of the initial concentration, respectively.

The breakthrough capacity (*q_b_*, mg/g) refers to the amount of Pb(II) adsorbed onto CCB at the breakthrough point. It is calculated using Equation (1) [50]:

(1) $$q_{b}=\int_{0}^{V_{b}}\frac{C_{t}-C_{o}}{m}dV$$

where *V_b_* refers to the total volume of treated effluent at the breakthrough point (mL), *m* is the adsorbent mass (g), and *C*_0_ (mg/L) and *C_t_* (mg/L) refer to the initial concentration and effluent concentration at any time *t*, respectively.

The exhaustion capacity (*q_e_*, mg/g) refers to the Pb(II) quantity removed by CCB at the exhaustion point. It is computed using Equation (2) [50]:

(2) $$q_{e}=\int_{0}^{V_{e}}\frac{C_{t}-C_{o}}{m}dV$$

where *V_e_* refers to the total volume of treated effluent at the breakthrough point (mL).

Under fixed-bed conditions, there is continuous transfer between solute (influent) and CCB. The mass transfer zone (*Z_m_*, cm) refers to the length in the CCB column where Pb(II) adsorption actively occurs. The length of the MTZ (*Z_m_*) is calculated from the breakthrough curve using Equation (3) [51]:

(3) $$Z_{m}=Z\left(1-\frac{t_{b}}{t_{e}}\right)$$

where *Z* is the bed height (cm), *t_b_* is the time of breakthrough (min) and *t_e_* is the time of exhaustion (min).

#### Fixed-Bed Adsorption Model

The experimental data of the adsorption of Pb(II) under fixed-bed conditions are defined using kinetic models. The breakthrough profiles are predicted using the Thomas model and the Clark model.

The Thomas model is one of the most widely used equations. The model assumes the following: (a) axial dispersion does not take place in the fixed bed, (b) adsorption-desorption follows the Langmuir equation, and (c) the adsorption process follows the second-order reversible reaction kinetics [52,53]. The linear form of the Thomas model is given in Equation (4):

(4) $$ln\left(\frac{C_{0}}{C_{t}}-1\right)=\frac{k_{Th}Q_{0}m}{Q}-\frac{k_{Th}C_{0}}{Q}V_{t}$$

where *V_t_* is the treated volume of effluent at any time *t* (mL), *Q* refers to influent flow rate (mg/L), *Q*_0_ is the maximum solid phase concentration of contaminant per weight of adsorbent (mg/g), and *k_Th_* is the Thomas rate constant (mL/mg·min).

The Clark model predicts the breakthrough curves based on the assumption of equilibrium adsorption and mass transfer. Other assumptions include: (a) Freundlich isotherm best describes the mass transfer in the fixed bed while (b) piston type refers to the flow of influent within the column [54]. The linear form of the model is given in Equation (5) [55]:

(5) $$ln\left({\left(\frac{C_{0}}{C_{t}}\right)}^{n-1}-1\right)=-rt+lnA$$

where *A* and *r* (min^−1^) are the Clark constants, and *n* refers to the Freundlich constant derived from batch experiments.

One of the simple fixed-bed models is the Yoon–Nelson model. The application of the model does not require information such as physical characteristics of the column bed, type of adsorbent, or physicochemical properties of the adsorbent [56]. The model is based on the assumption that the reduced rate of adsorption for each contaminant molecule is correlated to the probability to reach the breakthrough of the adsorbent bed and probability of adsorption of the contaminant [57]. The linear form of the Yoon–Nelson model is provided in Equation (6):

(6) $$ln\left(\frac{C_{t}}{C_{0}-C_{t}}\right)=K_{YN}t-K_{YN}\tau$$

where *τ* (min) is the time to reach 50% breakthrough and *K_YN_* (min^−1^) refers to the rate constant.

## 3. Results and Discussion

### 3.1. Characterization of CCB

**Figure 1:**
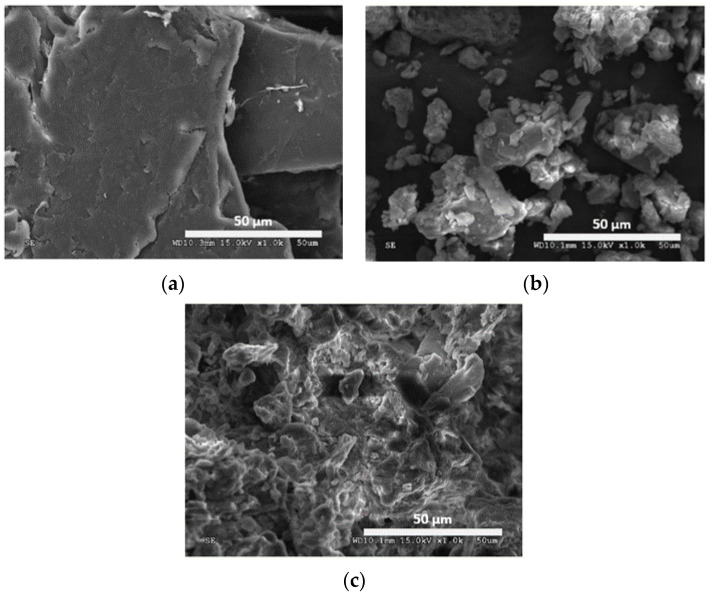
SEM micrographs of (**a**) chitosan, (**b**) bentonite, and (**c**) CCB.

The surface morphologies of chitosan, bentonite, and CCB are illustrated in Figure 1. Chitosan particles display a continuous, smooth surface and are composed of large particles with diameter greater than 100 µm, while bentonite is composed of smaller particles (ranging from 5 to 37 µm in diameter) with a rougher surface. The surface morphology of CCB shows an irregular surface and a more aggregated structure.

**Figure 2:**
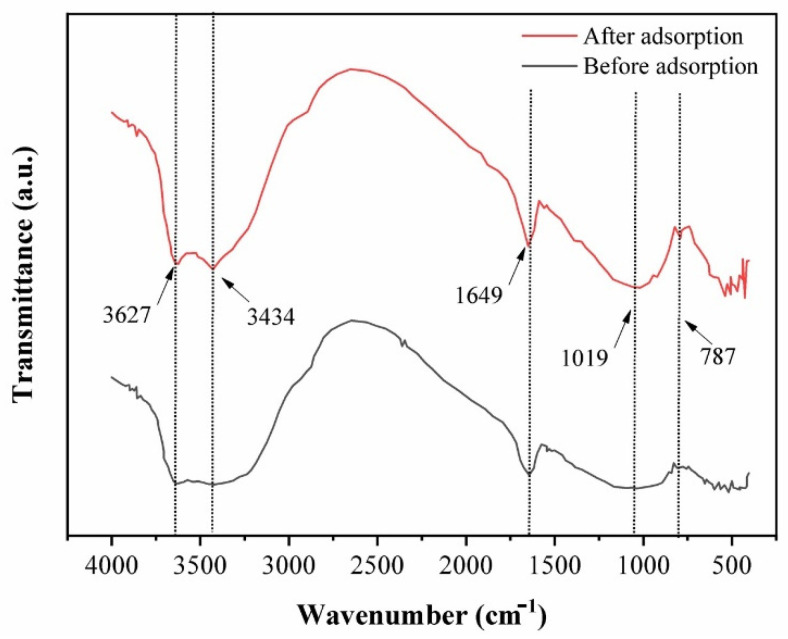
FT-IR spectra of CCB before and after adsorption with Pb(II).

The FT-IR spectra of raw CCB and CCB loaded with Pb(II) are shown in Figure 2. The following peaks are attributed to chitosan: 3434 cm^−1^ due to stretching of N-H and O-H groups and 1649 cm^−1^ due to amide I bands and C=O stretching of acetyl units [34,58]. Bentonite is represented by the following peaks: 787 cm^−1^ due to vibrations of Mg-OH and Al-OH, 1019 cm^−1^ due to stretching of Si-O groups, 1649 cm^−1^ due to O-H bending vibration, and 3434–3627 cm^−1^ due to O-H stretching vibrations [34,59,60]. After adsorption, the peaks at 3627 cm^−1^ and 3434 cm^−1^ become sharper, which implies interaction between CCB and Pb(II) via the O-H groups of bentonite [61].

### 3.2. Effect of Flow Rate

**Figure 3:**
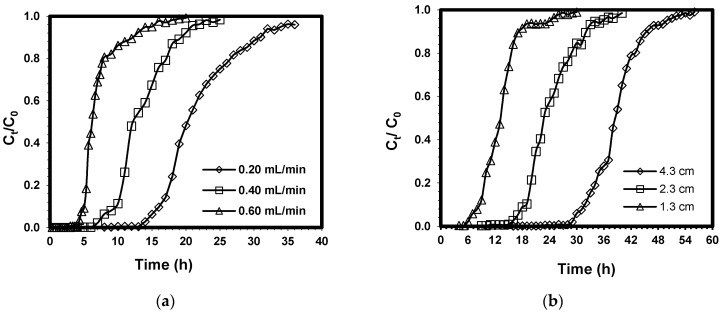

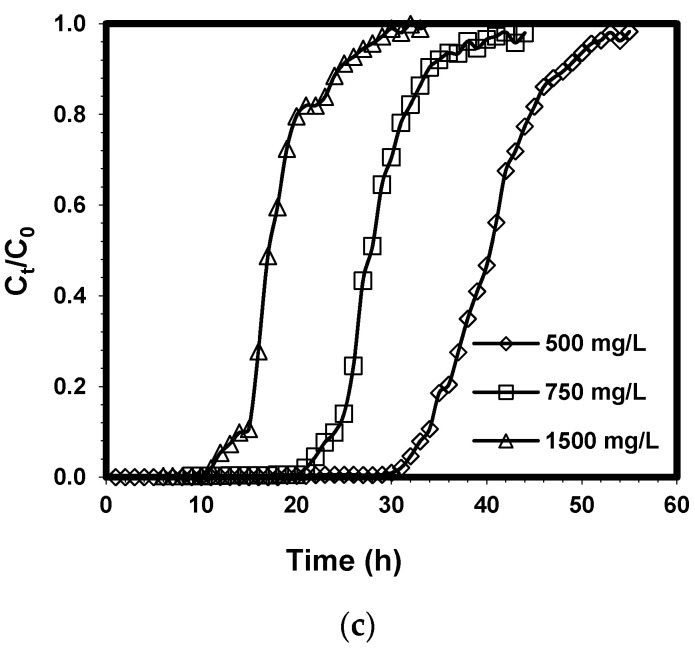
Effect of (**a**) flow rate, (**b**) bed height, and (**c**) initial Pb(II) concentration on the breakthrough capacity of fixed-bed CCB for the removal of Pb(II).

Figure 3a illustrates the breakthrough curves of Pb(II) under varying flow rates. The results show that breakthrough and exhaustion occurred earlier when the flow rate was increased from 0.20 mL/min to 0.60 mL/min. The breakthrough curves were observed to shift from left to right with a decrease in flow rate. At higher flow rates, breakthrough curves were observed to become steeper. Table 1 shows column parameters such as adsorption capacity at exhaustion (*q_e_*), adsorption capacity at breakthrough (*q_b_*), and length of MTZ (*Z_m_*). An increase in the value of *q_b_* and *q_e_* was observed as the flow rate was decreased from 0.60 to 0.20 mL/min. Meanwhile, low values of *Z_m_* were observed under low flow rate, indicating a decreasing length of MTZ. This means that low flow rate would cause a longer time for breakthrough and exhaustion in the fixed bed to occur due to an extended contact time for adsorption to take place since the MTZ movement along the fixed bed would be slower [62]. On the other hand, high values of flow rate mean limited time for diffusion of Pb(II) from the solute onto the binding sites located on the CCB surface and its pores, which would result in low values of *q_b_* and *q_e_*.

### 3.3. Effect of Bed Height

Figure 3b shows that increasing the bed height would result in an increase in the time to reach breakthrough and exhaustion. The breakthrough curve becomes sharper at lower bed heights, resulting in a quick exhaustion of the fixed bed. The curves take the appearance of a characteristic S-shape profile, which is an indication of ideal adsorption systems [63].

Table 1 shows that a higher bed height would result in high values of *q_b_* and *q_e_*. At bed depth of 4.3 cm, there is a higher amount of adsorbent present in the fixed bed, which implies that more binding sites are available for adsorption in the removal of Pb(II). In addition, bed depth of 4.3 cm would mean greater residence time of the influent solution within the CCB bed. At greater bed height, there is more contact time between Pb(II) and CCB particles; hence, there is enough time for Pb(II) ions to diffuse into the CCB bed for adsorption to occur. There was a slight increase observed for the length of MTZ, *Z_m_* as the bed height was increased from 1.3 to 4.3 cm.

### 3.4. Effect of Initial Concentration

Figure 3c illustrates the breakthrough curves of Pb(II) under different initial concentrations with constant flow rate and bed height of 0.20 mL/min and 4.3 cm, respectively. The time to reach breakthrough and exhaustion was observed to decrease as the initial concentration was increased from 500 to 1500 mg/L. At low initial concentrations, the breakthrough curves became more dispersed and the time to reach breakthrough was longer. When the initial concentration was increased to 1500 mg/L, the breakthrough curves became steeper, which indicates quick saturation of the fixed bed.

Based on Table 1, the values of *q_b_* and *q_e_* were observed to increase as the initial metal concentration was increased. Meanwhile, the value of *Z_m_* decreased as the initial concentration was reduced from 1500 mg/L to 500 mg/L. The main driving force of adsorption is the concentration gradient, which is the difference in concentration of Pb(II) on the CCB surface and Pb(II) in the solution [64]. A greater concentration difference provides a better driving force that helps in overcoming the mass transfer resistance, which results in high adsorption rates and better uptake of Pb(II) onto CCB. This explains why high *q_b_* and *q_e_* values were obtained at high initial concentrations.

### 3.5. Breakthrough Curve Modelling

To design a fixed-bed system, especially if implemented on an industrial scale, it is essential to evaluate and predict the breakthrough curves using different kinetic column models such as the Thomas and Clark models.

**Figure 4:**
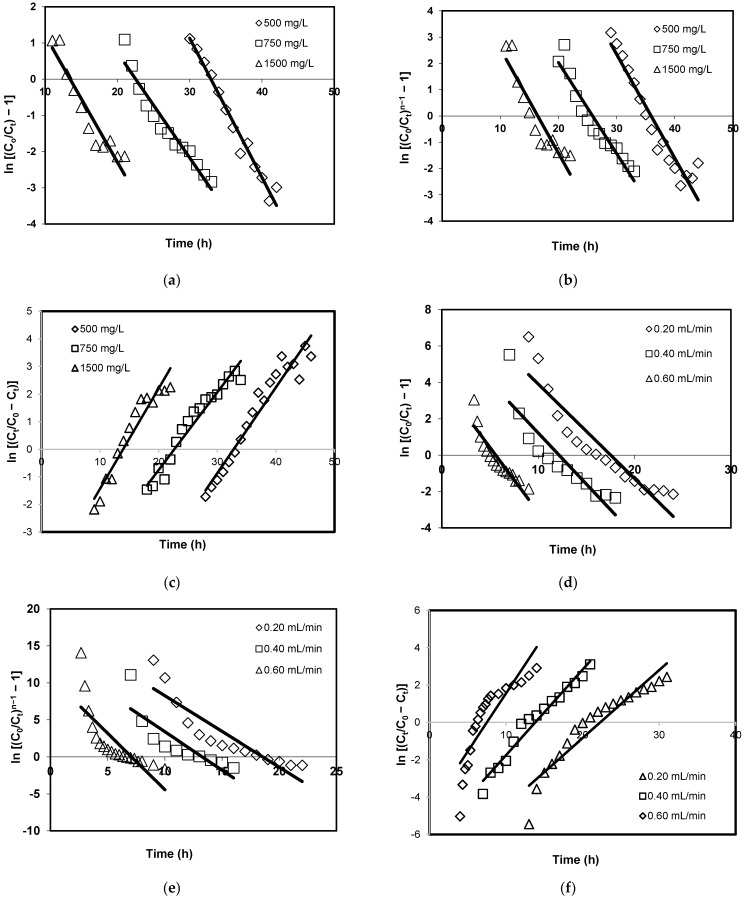
Adsorption of Pb(II) using CCB under a fixed-bed system under various initial concentrations fitted with the (**a**) Thomas model, (**b**) Clark model, and (**c**) Yoon–Nelson model. Effect of flow rate on the adsorption of Pb(II) using CCB under a fixed-bed system fitted with the (**d**) Thomas model, € Clark model, and (**f**) Yoon–Nelson model.

Table 2 displays the calculated parameters for the Thomas model under various flow rates and initial concentrations. The high values of the coefficient of determination, *R*^2^ (0.9758 ≤ *R*^2^ ≤ 0.8087), indicate that the Thomas model can be used to predict the breakthrough curves. In addition, high *R*^2^ values also imply excellent correlation between experimental data and theoretical linear data generated by the Thomas model. This is validated by Figure 4a,d, which display a good agreement between the theoretical linear plot from the Thomas model and experimental data under varying the flow rate and initial concentration. Overall, the Thomas model is a suitable fit in describing the adsorption of Pb(II) using CCB in a fixed-bed system. The results show that a higher flow rate causes the values of *k_Th_* to increase since mass transfer resistance is lower. Meanwhile, values of *Q*_0_ were observed to decrease at a high flow rate, which is attributed to the insufficient contact time between Pb(II) ions and binding sites on the surface of CCB. When the initial concentration was increased from 500 to 1500 mg/L, there was an increase in *Q*_0_ values observed due to a better concentration gradient, which is the driving force of adsorption [65]. Meanwhile, the *k_Th_* values were lower at high initial concentrations due to the effect of steric crowding. At 1500 mg/L, the repulsive forces between Pb(II) ions in the influent were stronger due to shorter distances between contaminants that would cause a longer time to achieve equilibrium. The trend observed in the present work is similar to the outcome observed by Dovi et al. [66] and Han et al. [67]. The values of *Q*_0_ obtained in the present work are higher in comparison to adsorption capacity values under batch conditions [37]. Under fixed-bed conditions, CCB is continuously in contact with a larger amount of wastewater. Moreover, the dynamic nature of a fixed-bed system may result in various retention mechanisms for Pb(II) aside from adsorption [68,69].

Under different initial concentrations and flow rates, Figure 4b,d demonstrate the experimental data fitted with linear plots generated by the Clark model while Figure 4c,f show the fitting of the Yoon–Nelson model with experimental data. Based on Table 2 and Table 3, the results show that lower *R*^2^ values were obtained for both the Clark model (0.6791 ≥ *R*^2^ ≥ 0.9228) and the Yoon–Nelson model (0.7407 ≥ *R*^2^ ≥ 0.9380). This indicates that the Clark and Yoon–Nelson models cannot adequately describe the breakthrough curves. In comparison to the Thomas model, the results also show that the linear plots derived from the Clark model and the Yoon–Nelson model are not in good correlation with the experimental data.

Table 4 summarizes the maximum adsorption capacity of numerous adsorbents investigated in previous studies on the removal of Pb(II) under a fixed-bed system. The adsorption capacity of CCB is lower when compared to that of intercalated kaolinite and clinoptilolite granules. The study of Tsai et al. (2016) showed the adsorption capacity of CCB in a multi-metal system (Cu(II), Ni(II), Pb(II)) to be lower when compared to the present study [40]. This implies tight competition for binding sites on the CCB surface among Cu(II), Pb(II), and Ni(II) in the aqueous solution. Overall, CCB has a satisfactory adsorption capacity in the removal of Pb(II), which suggests its potential to be utilized as a reactive material in a PRB system for the passive treatment of contaminated groundwater.

## 4. Conclusions

The present work examined the adsorptive removal of Pb(II) from an aqueous solution under a fixed-bed system. The impacts of different column parameters such as initial concentration, bed height, and flow rate on the fixed bed performance were determined. Breakthrough and exhaustion times were observed to increase with increasing bed height, decreasing flow rate, and low initial concentrations. Breakthrough curves became sharper under shorter bed height, high initial concentrations, and increasing flow rate. FT-IR analysis reveal that O-H groups of bentonite are involved in the adsorptive removal of Pb(II) from aqueous solution using CCB. Breakthrough curves were described by applying kinetic models such as the Clark, Yoon–Nelson, and Thomas models. Based on the high values of *R*^2^ (*R*^2^ ≤ 0.8087), the Thomas model was determined to be the best in predicting the breakthrough curves. There is a good agreement observed between the linear plots generated by the Thomas model and the experimental data. Overall, the results show the capacity of CCB under fixed-bed conditions in removing Pb(II) from aqueous solution on a laboratory scale. Further studies of the design parameters of the fixed-bed system using a pilot scale are recommended in preparation for its application in the industry. In the present work, a simple synthesis approach is provided in developing a chitosan–bentonite composite material that could be utilized in the removal of Pb(II) from industrial wastewaters and contaminated groundwater.

## Figures and Tables

**Table 1:** Length of mass transfer zone, adsorption capacity of Pb(II) at breakthrough, and adsorption capacity of Pb(II) at exhaustion under various operating conditions.

Parameter		*q_b_* (mg/g)	*q_e_* (mg/g)	*Z_m_* (cm)
Flow rate (mL/min) ^1^	0.20	22.81	30.03	0.93
	0.40	21.77	28.83	1.04
	0.60	20.95	28.46	1.40
Bed height (cm) ^2^	1.3	22.62	30.25	0.87
	2.3	24.69	34.36	0.98
	4.3	26.11	32.93	1.04
Initial concentration (mg/L) ^3^	500	24.03	28.34	1.19
	750	24.86	29.02	1.26
	1500	29.52	37.71	2.02

^1^ Bed height = 2.3 cm; initial concentration = 500 mg/L. ^2^ Flow rate = 0.20 mL/min, initial concentration = 500 mg/L. ^3^ Bed height = 4.3 cm; flow rate = 0.20 mL/min.

**Table 2:** Kinetic model constants of the Thomas model and the Clark model on the adsorptive removal of Pb(II) from aqueous solution using CCB.

*Q* (mL/min)	*Z* (cm)	*C*_0_ (mg/L)	Thomas			Clark		
			*k_Th_*	*Q*_0_	*R* ^2^	*A*	*r*	*R* ^2^
0.20	4.3	500	0.0008	32.93	0.9758	149.70 × 10^2^	0.3958	0.9204
0.20	4.3	750	0.0004	33.79	0.9402	77.20 × 10^2^	0.3460	0.9228
0.20	4.3	1500	0.0001	41.38	0.9054	681.64	0.2972	0.8657
0.20	2.3	500	0.0010	35.04	0.8689	594.11 × 10^2^	0.9645	0.8181
0.40	2.3	500	0.0011	33.77	0.8087	193.75 × 10^2^	1.0362	0.7139
0.60	2.3	500	0.0014	30.53	0.8380	480.98	1.5203	0.6791

**Table 3:** Kinetic model constants of Yoon–Nelson model on the adsorptive removal of Pb(II) from aqueous solution using CCB.

*Q* (mL/min)	*Z* (cm)	*C*_0_ (mg/L)	Yoon–Nelson		
			*K_YN_*	*τ*	*R* ^2^
0.20	4.3	500	0.3116	32.78	0.9380
0.20	4.3	750	0.2821	22.68	0.9012
0.20	4.3	1500	0.3634	13.93	0.8949
0.20	2.3	500	0.6214	7.51	0.7407
0.40	2.3	500	0.4577	13.83	0.8776
0.60	2.3	500	0.3637	22.31	0.8549

**Table 4:** Comparison of adsorption capacities of various adsorbents in the removal of Pb(II) using fixed-bed systems.

Adsorbents	Adsorption Capacity (mg/g)	References
Dead calcareous skeletons	38.46	[70]
Natural kaolinite	15.52	[71]
Chitosan-coated bentonite (multi-metal system)	13.49	[40]
Clinoptilolite granules	45.30	[72]
Sand-bone char	38.17	[73]
Intercalated kaolinite	52.55	[71]
Chitosan-coated bentonite (single metal system)	41.38	This study

## Data Availability

Data are within the article.
